# A dual propagation contours technique for semi-automated assessment of systolic and diastolic cardiac function by CMR

**DOI:** 10.1186/1532-429X-11-30

**Published:** 2009-08-13

**Authors:** Wei Feng, Hosakote Nagaraj, Himanshu Gupta, Steven G Lloyd, Inmaculada Aban, Gilbert J Perry, David A Calhoun, Louis J Dell'Italia, Thomas S Denney

**Affiliations:** 1 Electrical and Computer Engineering Department, Auburn University, Auburn, AL 36849, USA; 2 Division of Cardiovascular Disease, University of Alabama at Birmingham, Birmingham, AL 35294, USA; 3 Department of Biostatistics, University of Alabama at Birmingham, Birmingham, AL 35294, USA

## Abstract

**Background:**

Although cardiovascular magnetic resonance (CMR) is frequently performed to measure accurate LV volumes and ejection fractions, LV volume-time curves (VTC) derived ejection and filling rates are not routinely calculated due to lack of robust LV segmentation techniques. VTC derived peak filling rates can be used to accurately assess LV diastolic function, an important clinical parameter. We developed a novel geometry-independent dual-contour propagation technique, making use of LV endocardial contours manually drawn at end systole and end diastole, to compute VTC and measured LV ejection and filling rates in hypertensive patients and normal volunteers.

**Methods:**

39 normal volunteers and 49 hypertensive patients underwent CMR. LV contours were manually drawn on all time frames in 18 normal volunteers. The dual-contour propagation algorithm was used to propagate contours throughout the cardiac cycle. The results were compared to those obtained with single-contour propagation (using either end-diastolic or end-systolic contours) and commercially available software. We then used the dual-contour propagation technique to measure peak ejection rate (PER) and peak early diastolic and late diastolic filling rates (ePFR and aPFR) in all normal volunteers and hypertensive patients.

**Results:**

Compared to single-contour propagation methods and the commercial method, VTC by dual-contour propagation showed significantly better agreement with manually-derived VTC. Ejection and filling rates by dual-contour propagation agreed with manual (dual-contour – manual PER: -0.12 ± 0.08; ePFR: -0.07 ± 0.07; aPFR: 0.06 ± 0.03 EDV/s, all P = NS). However, the time for the manual method was ~4 hours per study versus ~7 minutes for dual-contour propagation. LV systolic function measured by LVEF and PER did not differ between normal volunteers and hypertensive patients. However, ePFR was lower in hypertensive patients vs. normal volunteers, while aPFR was higher, indicative of altered diastolic filling rates in hypertensive patients.

**Conclusion:**

Dual-propagated contours can accurately measure both systolic and diastolic volumetric indices that can be applied in a routine clinical CMR environment. With dual-contour propagation, the user interaction that is routinely performed to measure LVEF is leveraged to obtain additional clinically relevant parameters.

## Background

Temporal changes in left ventricular (LV) volume over the cardiac cycle provides fundamental information regarding systolic and diastolic function of the heart but is difficult to measure by standard clinical techniques. Cine cardiovascular magnetic resonance (CMR) using serial short axis slices is well accepted as a gold standard for measuring geometry-independent ventricular volumes [1,2]. Measurement of LV ED and ES volumes is based on drawing contours at the ED and ES time points. If contours could be reliably identified in all acquired time frames, ventricular volume-time curves (VTC) could be constructed, from which important parameters of ventricular function such as peak ejection rates (PER) and peak filling rates (PFR) [3,4] can be derived. These parameters may complement flow indices [5] in the assessment of diastolic function.

Fully automated contouring techniques have been a research topic for many years [6-15], and, more recently, techniques have been developed for propagating contours drawn at single time frame to the remaining time frames [16-22]. While the accuracy of these methods continues to improve, contour review and editing by a trained expert is still mandatory. A common problem encountered in myocardial contour identification is the presence of papillary muscles; following the echocardiographic convention, papillary muscles are often excluded from the endocardial contour. At ED in the short axis CMR image, papillary muscles are usually not a problem because they are separated from the LV wall. During systole, however, the papillary muscles move close to the LV wall, and it can be difficult to distinguish papillary muscles from the heart wall without carefully examining the images. For this reason, fully automatic contouring routines often have difficulty detecting papillary muscles, and may include papillary muscle volume as part of the LV cavity volume in ED and as outside the LV cavity (i.e., in the myocardial muscle volume) in ES. This potentially affects the derived volumes and masses.

Consequently, we propose a semi-automated method which leverages the user interaction in drawing ED and ES contours by automatically propagating them to all other time frames in a typical cardiac scan. This dual-contour propagation technique has the potential to more accurately exclude papillary muscles from the LV wall than single-contour propagation techniques or fully automated techniques. The proposed dual-contour propagation technique will not require additional work by the user, because at most institutions contours are already routinely drawn at ED and ES to compute standard volumes, myocardial mass, and ejection fraction. The purpose of this study was to develop a novel semi-automated technique using dual-contour propagation to measure ventricular volumes throughout the cardiac cycle, and compare this method to manual and single-contour techniques in normal volunteers and hypertensive patients.

## Methods

### Subjects

The study was approved by the appropriate institutional review boards and informed consent was obtained from all the participants. 39 normal human volunteers (NLs) and 49 hypertensive (HTN) patients consecutively enrolled in a study of resistant hypertension (defined as requiring 3 or more anti-hypertensive medications to achieve blood pressure < 140/90 mmHg) participated in this study. All patients were in sinus rhythm at the time of CMR.

### Image Acquisition

CMR was performed on a 1.5-T scanner (CV/i, GE Healthcare, Milwaukee, WI) optimized for cardiac application. ECG-gated, breath-hold steady state free precision technique was used to obtain standard (2, 3 and 4 Chamber, Short Axis) views using the following parameters – slice thickness 8 mm with no gap between short-axis slices, field-of-view 44 × 44 cm, scan matrix 256 × 128, flip angle 45°, typical TR/TE = 3.8/1.6 ms; typical acquired temporal resolution approximately 40 ms); data reconstructed to 20 cardiac phases.

### Image Analysis

In all scans, LVED and LVES endocardial contours were manually drawn on all short axis slices between the mitral annulus and apex [23] with exclusion of the papillary muscles. These contours were then automatically propagated to all the other frames in the acquisition using the dual-contour propagation algorithm described below. For validation, LV contours were manually drawn on all time frames in 18 randomly-selected normal scans by a Level 3 trained CMR specialist. These contours were used as a gold standard for evaluating and validating the dual-contour propagation algorithm.

### Contour Propagation

Non-rigid registration (NRR) [24] was used to propagate the contours manually drawn at end-diastole and end-systole to all other time frames in the acquisition. The NRR algorithm computed a deformation field that warped a source image to fit a template image. The deformation field was then used to propagate contours defined on the template image to the source image. Details of this algorithm are provided in the Appendix. All algorithms were implemented in MATLAB (The Mathworks, Natick, MA).

**Figure 1:**
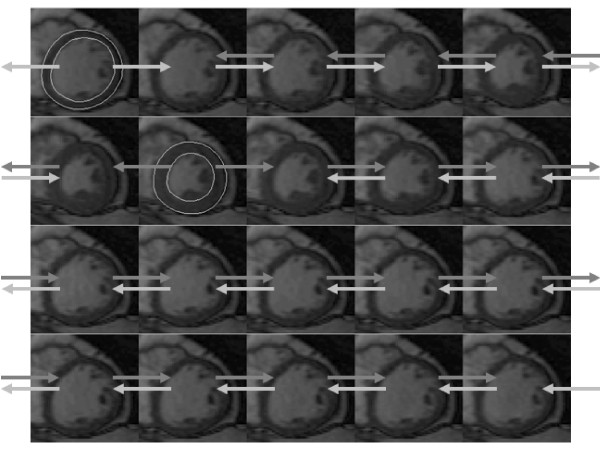
**Contour propagation strategy**. Manually-drawn contours at ED (top-left) and ES (2^nd ^row and 2^nd ^column) were propagated to other time frames and combined.

The dual-contour propagation scheme shown in Figure 1 was used to propagate both ED and ES contours to all other time frames in the sequence. First, the NRR algorithm was used to propagate ED contours forward in time through systole and backward in time through diastole (white arrows in Figure 1). Next, ES contours were propagated forward in time through diastole and backward in time through systole (gray arrows in Figure 1). The contours propagated from ED and ES were then combined, as described in the Appendix, into a single set of endocardial and epicardial contours.

### Volumetric Analysis

The LV volume at each time frame was computed by summing the volumes defined by the contours in each slice. The contour propagation procedure, however, propagated contours in all slices that were contoured at ED, and, near the base, the LV margin may have moved through the image plane in systole. To address this problem, the NRR algorithm was used in a long-axis slice to track a user-selected point near the mitral annulus through the image sequence. The displacement of this point was used to determine how much each short-axis slice should be included in the volume computation [4,25]. For example, if the mitral annulus displaced 12 mm between ED and the current time frame and the slice thickness was 8 mm, the most basal slice would not be used in the volume computation and 50% of the second-most-basal slice volume would be used in the total volume. This mitral annulus tracking procedure was used to compute volumes for all the contouring techniques discussed in this paper, including manual contouring and automatic contour propagation algorithms.

Once the volumes were computed in each time frame, a VTC curve was constructed and differentiated with respect to time. End-diastole was defined as the maximum-volume time frame, and end-systole was defined as the minimum-volume time frame. Early diastole and late diastole were defined as the first and second halves respectively of the diastolic interval. The peak ejection rate (PER) was defined as the maximum negative time derivative during the systolic interval. The early diastolic and late diastolic peak filling rates (ePFR and aPFR) were defined as the maximum derivative during the early and late diastole.

### Comparison Between Single and Dual-Contour Propagation

Most existing contour propagation techniques propagate contours from either ED or ES time frames [8,16,20-22,26]. Volumes computed from dual-propagated contours, single-propagated contours from ED and ES using the NRR method described above, and single-propagated contours from ED and ES using CAAS MRV for Windows, version 3.2 (Pie Medical Imaging, Maastricht, the Netherlands), software were compared to volumes computed from manual contours on nine randomly-selected normal studies (identified by NV1-NV9). A VTC was computed for each type of contours for each study. To compare VTCs computed from different types of contours, differences were computed at each time point by subtracting the manual volume from the propagated volumes.

### Inter-User Variability

To assess inter-user variability in volumes computed from propagated contours, nine normal studies (NV10-NV18) were randomly selected. For each study, two sets of contours were manually drawn at ED and ES by different users. Each user was a Level 3 trained CMR specialist or equivalent. Each set of ED and ES contours was propagated using the dual contour technique, and VTCs and ejection/filling rates were computed.

### LV Mass Evaluation

To evaluate stability of dual-propagated contours throughout the cardiac cycle, LV mass measurements were computed in each time frame for studies NV10-NV18 from dual-propagated contours and compared to that from one set of manually-drawn contours.

### Comparison of PER and PFR Values in Normals and Hypertensives

The dual-contour propagation algorithm was used to propagate contours to all time frames and compute VTCs and ejection/filling rates in all 39 normals and 49 hypertensives.

### Statistical Analysis

Comparisons of LV volumes computed from different propagation schemes and comparisons of LV masses computed from dual-propagated and manual contours were performed using mixed modeling via PROC MIXED (SAS version 9.1). To account for the repeated measures within a subject, a compound symmetry correlation structure was assumed. In the LV volume study, confidence intervals on the differences based on the fitted mixed model were constructed each at 99% level to achieve a joint confidence level of at least 95% for this set of confidence intervals using Bonferroni adjustment [27].

Comparisons of PER and PFR values computed from dual-propagated and manually-drawn contours and comparisons of contours propagated by two different users were performed using two-tailed paired t-tests, correlation analysis, and Bland-Altman analysis. PER and PFR values derived from dual-propagated contours in hypertensive patients were compared to normals using unpaired t-tests. In all these statistical tests, a P-value less than 5% was considered statistically significant.

## Results

### Comparison Between Single and Dual-Contour Propagation

**Figure 2:**
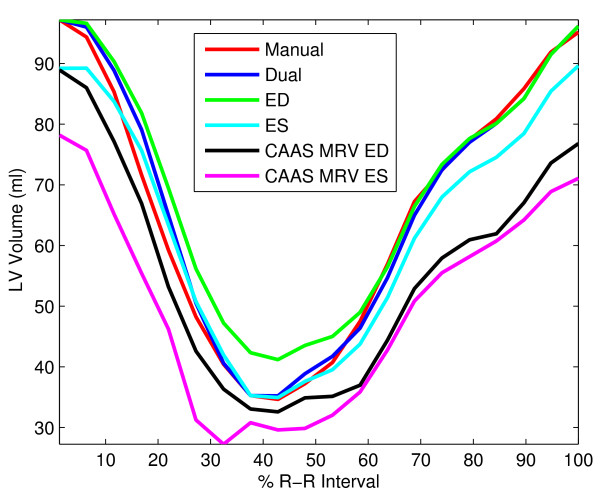
**LV-volume-versus-time curves for a normal human volunteer**. They were computed from different sets of contours: manually-drawn contours in each time frame (red), contours propagated from the manual ED contours using NRR (green), contours propagated from the manual ES contours using NRR (cyan), contours propagated from both ED and ES contours using NRR (blue), contours propagated from manual ED contours using CAAS MRV (black), and contours propagated from manual ES contours using CAAS MRV (magenta).

Differences between propagated contours and manual contours resulted in differences in VTCs. Figure 2 shows VTCs from a normal volunteer. ED-propagated contours with NRR resulted in volume overestimation near ES, and ES-propagated contours with NRR produced volume underestimation in early systole and late diastole. Dual-propagated contours showed excellent agreement throughout the entire cardiac cycle. Both ED and ES propagated contours using CAAS MRV underestimated the volumes as compared to the manually drawn, gold standard volumes, more than NRR propagated contours throughout the cardiac cycle; the CAAS MRV propagation method also changes the manually-drawn ED and ES contours slightly, so the volume difference is not zero at ED or ES in these curves.

Table 1 shows confidence intervals of the volume differences between each propagation method and manual. ED-propagated contours with NRR overestimate LV volume, whereas ES-propagated contours with NRR underestimate LV volume. In comparison, both ED-propagated and ES-propagated contours with CAAS MRV underestimate LV volume by a larger margin. However, the dual-propagated volumes were not statistically different from manually-contoured volumes.

**Table 1:** Differences between LV volumes (expressed as fraction of EDV) computed from propagated contours and manually-drawn contours.

	Volume Difference (EDV)			
		
	Mean ± SE	99% Confidence Interval		P
Dual NRR – Manual	-0.19 ± 0.56	-1.74	1.35	0.7316
ED NRR – Manual	1.61 ± 0.56	0.08	3.14	0.0069
ES NRR – Manual	-3.50 ± 0.56	-5.03	-1.97	<0.0001
ED CAAS – Manual	-6.54 ± 0.55	-8.06	-5.02	<0.0001
ES CAAS – Manual	-11.05 ± 0.55	-12.57	-9.54	<0.0001

SE = standard error.

The average computation time for dual-contour propagation (not including manually contouring the ED and ES contours) was 7.3 minutes for a single study on a 2.6 GHz dual-core personal computer with 4 GB of RAM. Automated contour propagation using CAAS MRV required less than 1 minute per study. Manual contouring of all slices and phases (typically 12 to 14 short axis slices × 20 cardiac phases) required approximately 4 hours per study.

### Validation of Functional Parameters

**Figure 3:**
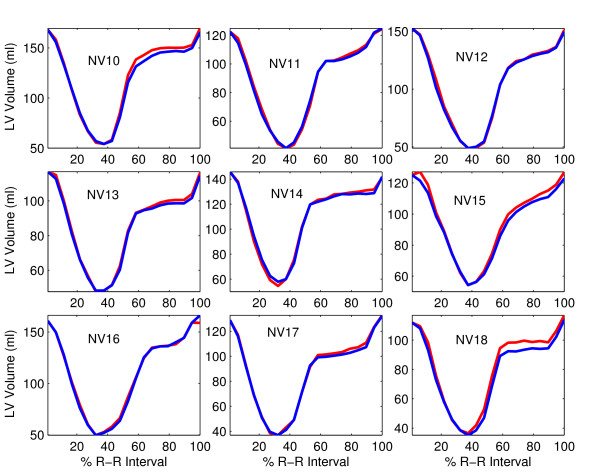
**LV-volume-versus-time curves for nine normal human volunteers**. They were computed from two different sets of contours: manually-drawn contours in each time frame (red) and dual-propagated contours (blue).

Figure 3 shows the VTCs in normal volunteers derived from the manually-drawn contours and dual-propagated contours. The manual and dual-propagated VTCs were quite close to each other in all nine studies – particularly during systole and early diastole. This similarity between manual and propagated VTCs means that the contours manually drawn at ED and ES were consistently propagated to the other time frames in the cine sequence.

**Figure 4:**
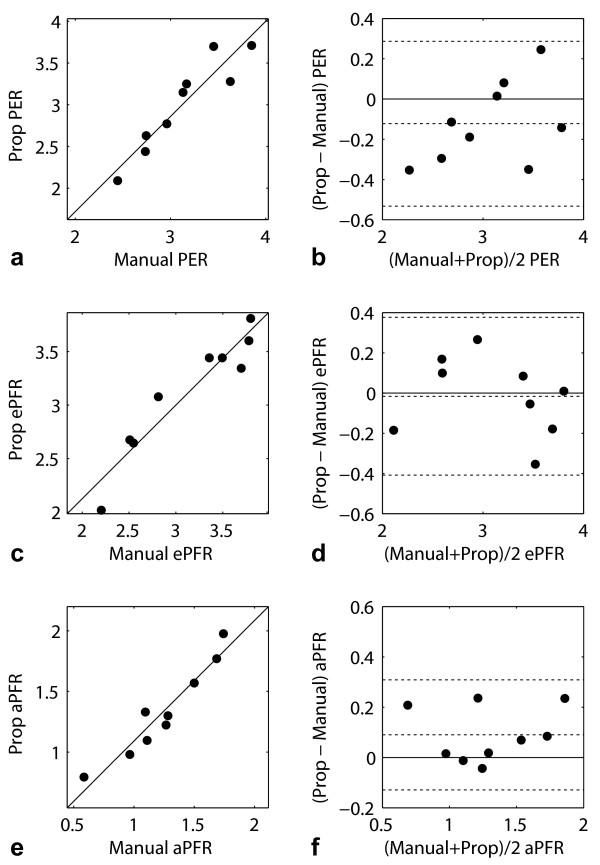
**Scatter and Bland-Altman plots of LV ejection and filling rates computed from manual contours (Manual) and dual-propagated contours (Prop)**. The plotted rates include peak ejection rate (PER) (a, b), early diastolic filling rate (ePFR) (c, d), and late diastolic filling rate (aPFR) (e, f) values in end-diastolic volumes (EDV)/sec. The dashed lines in Bland-Altman plots represent the mean and mean ± two standard deviations of the difference between Prop and Manual rates.

No statistically-significant differences were found between PER, ePFR and aPFR rates computed from manually-drawn contours and dual-propagated contours (Table 2). The correlation coefficients between the PER, ePFR and aPFR values were 0.92, 0.95 and 0.96 respectively (all P < 0.001). Figure 4 shows scatter and Bland-Altman plots comparing the manual and dual-propagated measurements of ejection and filling rates.

**Table 2:** Differences between peak ejection and filling rates computed from manually-drawn and dual-propagated contours.

	Rate Difference (EDV/s)			
		
	Mean ± SE	95% Confidence Interval		P
PER	-0.12 ± 0.08	-0.29	0.06	0.16
ePFR	-0.07 ± 0.07	-0.23	0.08	0.31
aPFR	0.06 ± 0.03	-0.02	0.13	0.11

Differences are dual-propagated minus manual.

PER: peak ejection rate; ePFR: early diastolic filling rate; aPFR: late diastolic filling rate; SE: standard error.

### Inter-User Variability

**Figure 5:**
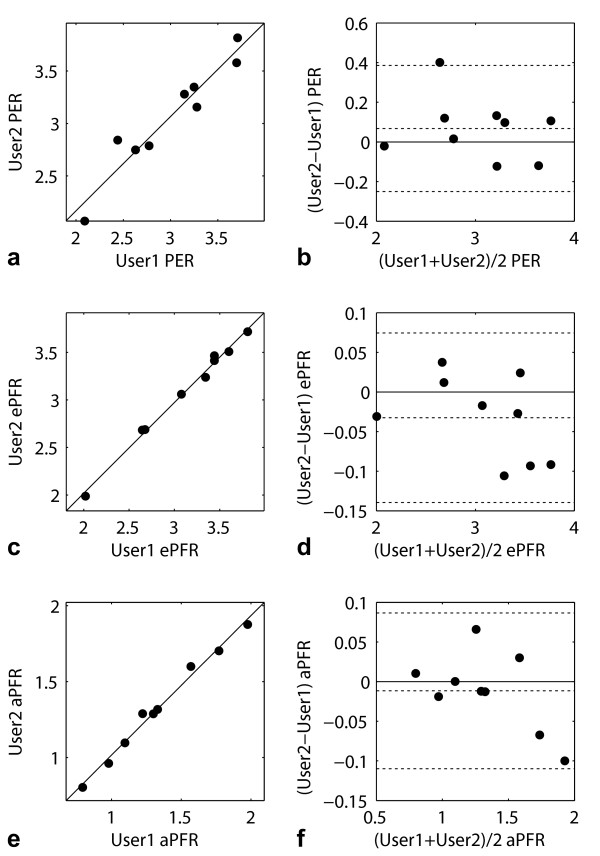
**Scatter and Bland-Altman plots of LV PER (a,b), ePFR (c,d), and aPFR (e,f) values in EDV/sec computed from contours manually drawn at ED and ES by two different users (User1 and User2) and propagated**.

No significant difference was found between PER, ePFR and aPFR values computed from contours propagated with ED and ES contours drawn by two different users (User1 and User2). The differences (User2-User1) between PER, ePFR and aPFR values were 0.07 ± 0.16 EDV/s (P = 0.24), -0.03 ± 0.05 EDV/s (P = 0.11) and -0.01 ± 0.05 EDV/s (P = 0.50) respectively. The correlation coefficients for the PER, ePFR and aPFR values were 0.95 (P < 0.0001), 0.99 (P < 0.0001) and 0.99 (P < 0.0001) respectively. Figure 5 shows scatter and Bland-Altman plots comparing User1 and User2 measurements of PER, ePFR and aPFR.

### LV Mass Evaluation

**Figure 6:**
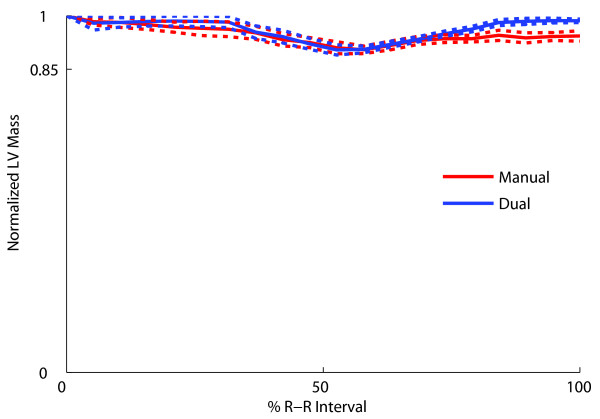
**Mean LV mass (normalized to ED LV mass) at different phases of the cardiac cycle computed from manual contours and dual-propagated contours averaged over nine normal studies**.

No significant difference was found between normalized LV masses (LV mass/ED LV mass) computed from dual-propagated contours and manual contours. The normalized LV mass difference (propagated-manual) was -0.015 ± 0.0077 (P = 0.08). Figure 6 shows the mean normalized LV mass throughout the cardiac cycle averaged over studies NV10-NV18. The normalized LV mass computed from dual-propagated contours was close to that from the manual contours and both remain stable throughout the cardiac cycle, indicating that the propagated contours were as stable as the manual contours.

### Peak Ejection and Filling Rates in HTN

**Figure 7:**
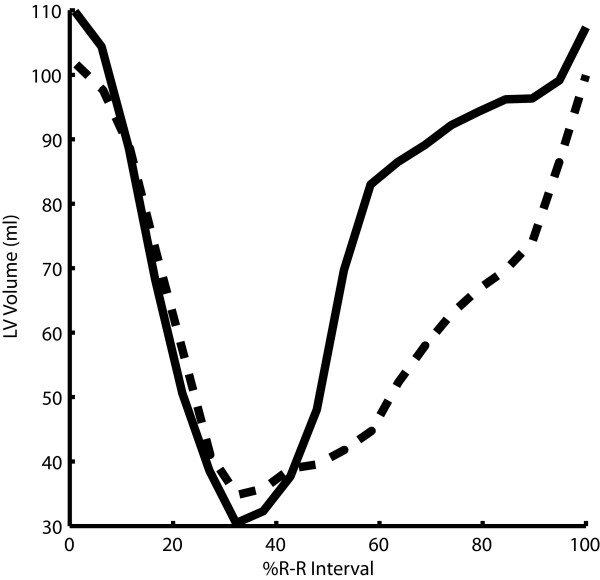
**Volume versus time (VTC) plots computed from dual-propagated contours for a normal volunteer (solid) and a hypertensive patient (dashed)**.

Figure 7 shows typical VTCs for a normal volunteer and a hypertensive patient measured from dual-propagated contours. Peak ejection rates are similar in both curves, but the early diastolic filling rate is lower in the hypertensive patient than in the normal while the late filling rate is higher.

**Figure 8:**
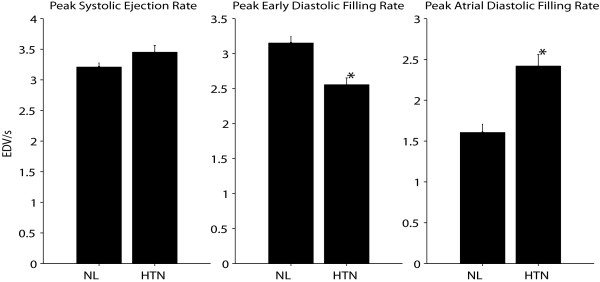
**Peak LV ejection rate, early diastolic filling rate, and late diastolic filling rate in EDV/s in normal volunteers (NL) and patients with primary hypertension (HTN)**. * P < 0.05 vs. normal.

Figure 8 shows the mean peak ejection and filling rates measured from dual-propagated contours in all 39 normals and 49 patients with hypertension. In hypertensives, PER was not different from normal (3.4 ± 0.1 vs. 3.2 ± 0.1 EDV/sec, P = NS). Diastolic filling rates, however, were altered compared to normals, demonstrating diastolic dysfunction in hypertension that is common in this patient group: ePFR was lower than normal (2.6 ± 0.1 vs. 3.2 ± 0.1 EDV/sec, P < 0.0001), but aPFR was higher than normal (2.4 ± 0.1 vs. 1.6 ± 0.1 EDV/sec, P < 0.0001).

## Discussion

In this paper, we described a novel dual-contour propagation technique for measuring volume-time curves (VTCs), validated it against manually drawn contours, and demonstrated its utility in a clinically-relevant patient population. This method requires nothing more than standard short-axis and long-axis CMR acquisitions and routinely drawn ED and ES contours. We show that the dual-propagated contours can be used to accurately measure peak ejection and filling rates compared to the reference standard of manually-drawn contours. The dual-contour propagation technique provides a fast, practical means of measuring volume-based indices of systolic and diastolic ventricular function from routine clinical CMR.

Several techniques have been proposed for propagating contours in CMR [16-22] and other modalities [28-33], but these techniques only propagate contours from a single time frame. While propagating contours from only one time frame requires less user interaction, we found that the resulting volumes are less accurate compared to dual propagation. If contours are only defined at ED, propagated contours with NRR may not be able to separate papillary muscles from the LV wall at ES resulting in the volume differences at ES (Figure 2). Propagating only ES contours with NRR may solve this problem, but volume differences occur in late diastole as demonstrated in Figure 2.

van Guens, et al. [15] proposed an automated method for drawing contours at ED and ES. The required user input was minimal – only four manually-drawn epicardial contours on two and four-chamber views at ED and ES – but volumes were only validated at ED and ES. In addition, for registration purposes, this method requires that both long axis and multiple short-axis acquisitions be performed with reproducible breath-hold positions, which can sometimes be difficult to obtain under clinical conditions. The contour propagation method proposed in this paper, however, does not have this limitation since contours are propagated in each slice independently.

Investigators have previously described use of volume time indices for measuring systolic and diastolic function [34,35]. CMR allows measurement of ventricular volumes throughout the cardiac cycle independent of geometric assumptions. The excellent spatial resolution and image contrast make it potentially the most accurate clinically-applicable non-invasive technique for assessment of systolic and diastolic function. To provide an illustration of the utility of our propagation method in clinical assessment of patients with risk factors for heart failure, the dual-contour propagation technique was employed to assess the physiology of LV systolic and diastolic function in 49 patients consecutively enrolled in a study of resistant hypertension. The images in this study contain the normal range of image quality and presence of artifacts encountered under routine clinical conditions. The concentrically hypertrophied LV in the HTN patients had a normal LV ejection fraction and LV peak ejection rate; however, early peak filling was decreased and late filling rate was increased, consistent with diastolic dysfunction.

The inter-user and intra-user variability in the propagated contours depend on the inter-user and intra-user variability of the semi-automatically-drawn contours at ED and ES. This variability has been studied in [15,36,37]. In our study, no significant difference was found between PER, ePFR and aPFR values computed from contours propagated from ED and ES contours drawn by two different users.

A limitation of contour propagation algorithms in general is that any errors in the seed contours get propagated to all other time frames. Consequently, it is especially important to ensure accurate seed contours before propagation. Also in this paper, papillary muscle volume was considered part of the LV blood volume. Since the papillary muscle volume is relatively constant throughout the cardiac cycle, subtracting the papillary muscle volume would reduce the blood volume by the same amount in all phases and would not significantly affect filling or ejection rates, which are the key parameters determined in this work. Although not evaluated in the present study, we believe that another potential advantage of the dual-contour propagation approach is that defining contours at two time points provides increased robustness to imaging artifacts.

In conclusion, the dual-contour propagation technique provides a fast, accurate and practical means of measuring volume-based indices of systolic and diastolic ventricular function from routine clinical CMR.

## Competing interests

The authors declare that they have no competing interests.

## Authors' contributions

WF developed the dual-contour propagation algorithm, drew one set of contours for the inter-user variability study, and drafted the manuscript. HN performed manual contouring of normal datasets including one set of contours for the inter-user variability analysis. HG participated in the study design and helped draft the manuscript. SGL participated in the study design and helped draft the manuscript. IA participated in the study design and performed the statistical analysis. GJP helped draft the manuscript. DAC participated in study coordination. LJD participated in study coordination and helped draft the manuscript. TSD conceived of the study, participated in study design and coordination and helped draft the manuscript. All authors read and approved the final manuscript.

## Appendix

### Non-Rigid Registration

First, a square region of interest (ROI) was defined that enclosed epicardial contours in the template image. Since the NRR was applied sequentially starting from either ED or ES, an epicardial contour (either manually-drawn or propagated) was always available in the template image.

Next, a two-dimensional displacement field was defined on the template ROI. The deformation field was parameterized by a tensor product of quadratic, uniformly-spaced B-splines:



where **p**is a point in the template ROI, **β**(**p**) is a B-spline basis function, **μ**_*i *_is a control point, *C *is the number of control points and **v**_*i *_is the knot location associated with the *i-*th control point. Eight control points were used in each dimension. The B-spline order and number of control points were determined empirically.

The B-spline control points, **μ**, were computed to minimize the sum of squared differences between the pixel intensities in the template image, *I*_*t*_, and the source image, *I*_*s*_:

(1)

where Ω is the set of pixels in the template ROI.

The cost function in Eq. (1) was optimized using a multi-resolution strategy to speed up computation and avoid local minima in the cost function. First, the template ROI was resampled from 64 × 64 to 16 × 16 pixels and the number of control points was reduced to four in each dimension. The reduced resolution control points were then computed using Levenberg optimization algorithm with an analytical gradient and Hessian. This process was then repeated using template ROI resampled from 64 × 64 to 32 × 32 pixels. The number of control points was kept at four in each dimension, and the result of the coarser resolution optimization was used as the starting point for the optimization. Next, the displacement field was interpolated to eight control points in each dimension, and Eq. (1) was optimized on the full-resolution 64 × 64 template ROI. The source image was resampled accordingly in each multi-resolution layer to match the resolution of the template ROI. The result was a spatially continuous mapping of points from the template ROI to the source image. Finally, each contour point in the template ROI was mapped to the source image using the final displacement field.

### Combining Contours Propagated From ED and ES

As described above, the NRR was used to propagate contours from both ED and ES to all other time frames in a sequence. These propagations resulted in two contours for each time frame (except at ED and ES). The two contours were combined into a single B-spline contour using a weighted-least-squares fit. The ED-propagated contour weight for a given time was computed using cubic-spline interpolation from the empirically-determined control points in Table 3. The end-systolic ES-propagated contour weight is one minus the ED contour weight. The ED-propagated and ES-propagated weights at a given frame are based on their distances from the ED and ES frames. For example, as the distance of a frame from ED increases, its ED-propagated weights decreases and its ES-propagated weights increases.

**Table 3:** Control points for generating ED-propagated contour weights.

% Systolic Interval	ED – Propagated Contour Weight	% Diastolic Interval	ED-Propagated Contour Weight
0.00	1.00	0.00	0.00
16.67	0.90	7.69	0.10
33.33	0.75	15.38	0.25
50.00	0.50	23.08	0.40
66.67	0.25	30.77	0.50
83.33	0.10	38.46	0.65
100.00	0.00	46.15	0.75
		53.85	0.85
		61.54	0.90
		69.23	0.95
		76.92	1.00
		84.62	1.00
		92.31	1.00
		100.00	1.00

ES-propagated contour weights are one minus the ED-propagated weights.
